# Rating of physiotherapy student clinical performance: is it possible to gain assessor consistency?

**DOI:** 10.1186/s12909-019-1459-4

**Published:** 2019-01-24

**Authors:** Garry W. Kirwan, Courtney R. Clark, Megan Dalton

**Affiliations:** 1 Physiotherapy Department, QEII Jubilee Hospital, Metro South Health, Coopers Plains, QLD 4109 Australia; 2 Menzies Health Institute, School of Allied Health Sciences, Griffith University, Gold Coast Campus, Southport, 4222 Australia; 3 School of Physiotherapy, Australian Catholic University, Sydney, Australia

**Keywords:** Physiotherapy, Consensus, Assessment, Students, APP

## Abstract

**Background:**

Reliable interpretation of the Assessment of Physiotherapy Practice (APP) tool is necessary for consistent assessment of physiotherapy students in the clinical setting. However, since the APP was implemented, no study has reassessed how consistently a student performance is evaluated against the threshold standards. Therefore, the primary aim of this study was to determine the consistency among physiotherapy educators when assessing a student performance using the APP tool.

**Methods:**

Physiotherapists (*n* = 153) from Australia with a minimum 3 years clinical experience and who had supervised a physiotherapy student within the past 12-months were recruited. Three levels of performance (not adequate, adequate, good/excellent) were scripted and filmed across outpatient musculoskeletal, neurorehabilitation, cardiorespiratory and inpatient musculoskeletal. In the initial phase of the study, scripts were written by academic staff and reviewed by an expert panel (*n* = 8) to ensure face and content validity as well as clinical relevance prior to filming. In the second phase of the study, pilot testing of the vignettes was performed by clinical academics (*n* = 16) from Australian universities to confirm the validity of each vignette. In the final phase, study participants reviewed one randomly allocated vignette, in their nominated clinical area and rated the student performance including a rationale for their decision. Participants were blinded to the performance level. Percentage agreement between participants was calculated for each vignette with an a priori percentage agreement of 75% considered acceptable.

**Results:**

Consensus among educators across all areas was observed when assessing a performance at either the ‘not adequate’ (97%) or the ‘good/excellent’ level (89%). When assessing a student at the ‘adequate’ level, consensus reduced to 43%. Similarly, consensus amongst the ‘not adequate’ and ‘good/excellent’ ranged from 83 to 100% across each clinical area; while agreement was between 33 and 46% for the ‘adequate’ level. Percent agreement between clinical educators was 89% when differentiating ‘not adequate’ from ‘adequate’ or better.

**Conclusion:**

Consistency is achievable for ‘not adequate’ and ‘good/excellent’ performances, although, variability exists at an adequate level. Consistency remained when differentiating an ‘adequate’ or better from a ‘not adequate’ performance.

## Background

In 1990, psychologist George Miller proposed a pyramid of hierarchy in the assessment of clinical competence. The levels ranged from knows, knows how (competence), shows how and does (performance) [1]. Within health professional programs such as physiotherapy, direct assessment of authentic clinical practice at the ‘does’ level is required to certify fitness to practice. That helps to assure relevant accreditation bodies, registration authorities and the broader population that graduates have met the required standards to safely and effectively practice within their particular health discipline [2]. Students are required to complete workplace based assessments in conjunction with their academic assessment as part of that credentialing process. Given the high stakes of these workplace based performance assessments, it is essential that the assessment practices are valid, reliable and fair to all students [3, 4].

Reliability of clinical assessment is the extent to which assessment yields consistent outcomes. During workplace based clinical placements, a student should expect a level of consistency between the assessors when rating their performance. While consistency in assessment is a reasonable expectation, there is a limited amount of research investigating this construct [5].

In physiotherapy programs across Australia and New Zealand, students are assessed on their competence to deliver entry-level physiotherapy services through completion of multiple longitudinal professional practice placements, commonly referred to as ‘clinical placements’. The passing or minimally competent standard is defined within the Physiotherapy Practice Threshold Statements [6]. Across Australia and New Zealand, The Assessment of Physiotherapy Practice (APP) instrument is used in most programs as the measure for assessing student performance against the entry-level standard [5, 7]. Aligned to the threshold standards, 20 items divided into seven domains of practice (professional behavior, communication, assessment, analysis and planning, intervention, evidence based practice and risk management) are assessed on a 5-point scale (0–4), where a score of two is defined as the minimally competent standard to enter the profession. In addition, overall performance is evaluated using a global rating scale (GRS) defined by four distinct categories to differentiate a student’s overall performance, namely, not adequate, adequate, good and excellent. Rasch analysis has shown the APP to be both reliable and valid in measuring changes in physiotherapy student competence over time [5, 7].

The APP was introduced in 2009 with empirical evidence to demonstrate strong validity and reliability among educators. However, more recently there has been anecdotal evidence suggesting a perceived variability in how educators interpret the APP. A recent study by Trede and Smith [8] supported this sentiment, demonstrating assessment practices among physiotherapy educators still relied on subjective factors despite their knowledge of the APP. It was observed that assessment practices were predominantly learnt in the workplace with little to no guidance on how to assess students despite training being available to educators [8]. Therefore, it is plausible that over time such practices have led to variability in the interpretation of the APP.

Reliable interpretation of the APP is necessary for consistent assessment of physiotherapy students in the clinical setting. Furthermore, physiotherapy programs and educators have a responsibility to ensure graduates meet the threshold standards by differentiating an inadequate (failing) performance from an adequate performance to ensure an appropriate standard of graduates are entering the profession.

Since the implementation of the APP, no study has assessed how consistent student performance is evaluated against the threshold standards. Therefore, the primary aim of the study was to determine what level of consistency is achieved between physiotherapy educators when assessing a student’s performance via video footage using the global rating scale of the APP. Secondly, the study also aimed to identify key attributes that influence educator decisions when applying the global rating scale of the APP.

## Method

A study was undertaken to determine consistency among physiotherapy clinical educators when rating videos of student performance using the GRS component of the APP. The Griffith University Human Ethics Committee granted ethical approval prior to the research commencing on 26th June 2014.

**Fig. 1:**
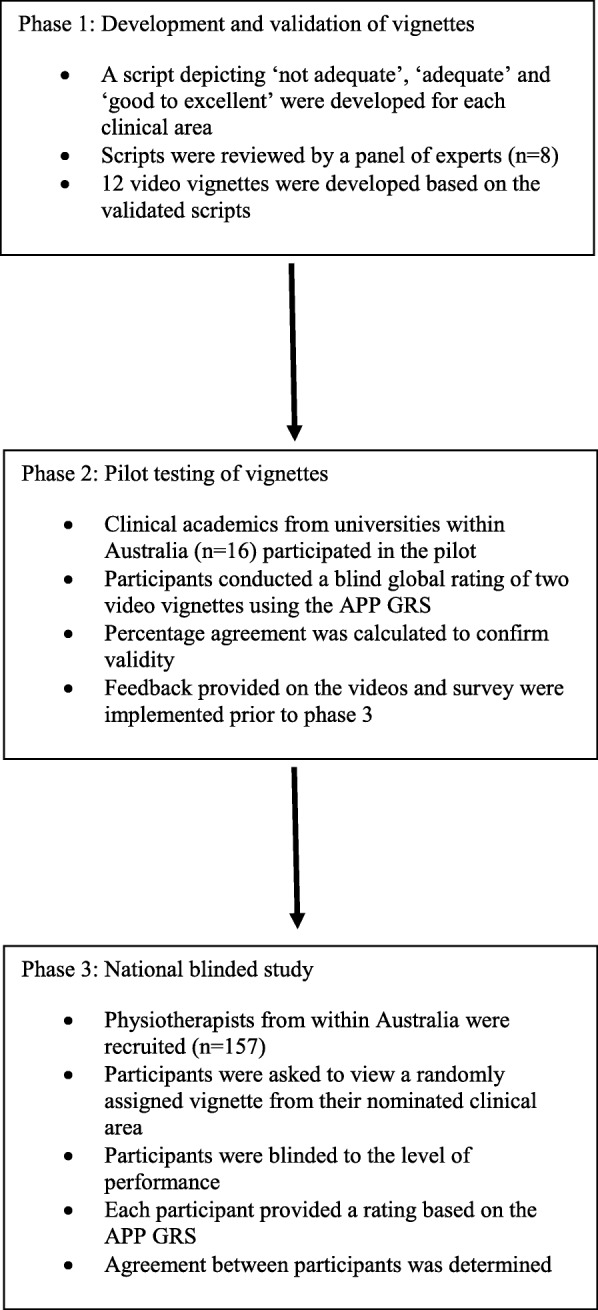
Study Design Flow Diagram

Four clinical scenarios were developed in the areas of inpatient musculoskeletal (Orthopaedics), outpatient musculoskeletal, cardiopulmonary and neurological physiotherapy to simulate a student performance. The study was divided into three phases as outlined in Fig. 1.

### Validation of the vignettes

Initially, each scenario from the four clinical areas were adapted into three separate scripts representing a ‘not adequate’, ‘adequate’ and ‘good/excellent’ performance based on the APP GRS. A panel of clinical educators (*n* = 8), experienced in each of the four clinical areas was convened to review the 12 scripts. Feedback was provided on the clinical authenticity and accuracy of the script and recommendations to improve face and content validity were implemented.

Once agreement was reached with all 12 scripts, each video was filmed using a standardised patient actor and an actor to portray the student physiotherapist. Academic staff from Griffith University played the role of clinical educator. During the filming of each scenario, the authors were present to direct each scene to ensure adherence to the scripts.

### Pilot testing of the video vignettes

A pilot study was undertaken using the final edit of each video. Clinical academics (*n* = 16), from across Australia, experienced in applying and interpreting the APP, were recruited to evaluate each video. Each participant watched two randomly selected videos, with the level of performance for each video blinded to the assessor. At the completion of each video, participants were instructed to provide a rating of the student’s performance based on the APP GRS. Participant responses were then compared to the scripted rating of performance to confirm the validity of each performance.

### Assessing consensus among physiotherapy clinical educators

Participants were recruited through an open invitation that was distributed through relevant national physiotherapy educator networks. An email containing details of the study and the relevant inclusion criteria was distributed. The inclusion criteria for the study was at least three year’s clinical experience, a minimum of one year’s experience in supervising physiotherapy students and each participant must have undertaken the primary supervision of at least one student in the past 12 months. Participants who did not meet the inclusion criteria were excluded to ensure the sample population was familiar with the assessment of student performance using the APP. Recipients of the email who met the inclusion criteria were asked to volunteer by contacting the research team in an ‘opt in’ model of recruitment. To categorise participants by clinical stream, volunteers were required to nominate the clinical area in which they felt most confident to assess student performance.

Once recruited, participants were sent an email containing instructions on completing the blind assessment. Videos were assigned using a Criterion-i purposive sampling method, as described by Palinkas, Horwitz, Green, Wisdom, Duan and Hoagwood [9]. This approach was adopted to ensure the ‘adequate’ performance was most viewed, as this was considered the critical decision when assessing student performance. A link to the allocated video from the clinical area nominated and a link to a survey hosted on www.SurveyMonkey.com was provided. Participants were instructed to watch the video vignette, rate the student’s performance using the APP GRS and provide three to five behaviours demonstrated by the student that most supported their rating decision. Furthermore, demographic data was collected for each participant.

### Data analysis

Relevant data was extracted from SurveyMonkey™ and divided into qualitative and quantitative data. Quantitative analysis was completed using SPSS 21.0 software package**®** (SPSS Inc., Chicago, IL, USA) and qualitative analysis and graphical representations were performed using Microsoft Excel**®** v2011 for Mac (Microsoft Corporation, Redmond WA).

Exact percentage agreement between respondents was calculated. Participant responses were also compared to the scripted level of performance of the video. Furthermore, the ability for an educator to differentiate between a ‘not adequate’ and ‘adequate’ performance was calculated. An a priori agreement of 75% was considered acceptable for the purposes of the study [7]. Analysis was conducted across all videos and by clinical area. Frequency and descriptive statistics were reported to identify demographic data about the sample population.

Thematic analysis was conducted on the qualitative data to identify common behaviours that most influenced clinical educator rating decisions regarding the student performance. Thematic data was divided by clinical area and mapped to one of the seven domains of the APP (professional behavior, communication, assessment, analysis and planning, intervention, evidence based practice and risk management).

## Results

### Participants

**Fig. 2:**
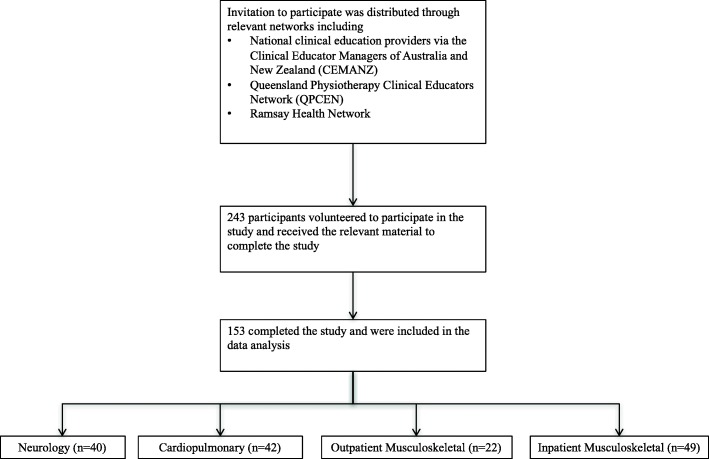
Participant Flow Diagram

Following the initial mail out, a sample of 243 participants, who met the inclusion criteria, volunteered to participate in the study. One hundred and sixty-seven participants participants responded to the survey (69%) and 153 completed all required components (63%). Participant recruitment and distribution by clinical area is outlined in Fig. 2.

Participant demographics are outlined in Table 1, showing a distribution across years of clinical and educational experience. In addition, all participants reported being at least somewhat confident in using the APP.

**Table 1:** Participant characteristics

Participant Demographics	Frequency (n)	Percent (%)
Clinical Experience (*n* = 166)		
3–5 years	44	26.5
6–8 years	54	32.5
9–11 years	25	15.1
12–15 years	16	9.6
More than 15 years	27	16.3
Experience as an Educator (*n* = 167)		
1–3 years	52	31.1
4–6 years	64	38.3
7–9 years	18	10.8
10–12 years	13	7.8
12–14 years	9	5.4
More than 14 years	11	6.6
Confidence in using the APP (n = 166)		
Not confident	0	0
Somewhat confident	39	23.5
Confident	108	65.1
Very confident	19	11.4

*APP* Assessment of Physiotherapy Practice

Six out of the eight Australian states and territories were represented in the study with the majority of respondents from Queensland (62%), Victoria (45%) and Tasmania (36%). Various clinical settings were represented in the sample including public (87%), community (14%) and private (4%). The three broad geographical regions were also represented in the sample including metropolitan (72%), regional (23%) and rural (4.8%).

### Consensus in rating student performance

Exact percentage agreement between clinical educators is outlined in Table 2. Strong consensus among educators across all videos was observed when assessing a student performing at either the ‘not adequate’ level (97%) or the ‘good/excellent’ level (89%). However, when assessing a student at the ‘adequate’ level, consensus among educators reduced to 43%. A similar trend was noted when student performance was split into the different clinical areas with consensus amongst the ‘not adequate’ and ‘good/excellent’ ranging from 83 to 100% across each clinical area; while agreement was between 33 and 46% for the ‘adequate’ level.

**Table 2:** Exact agreement between the proposed level of performance depicted by the video scenario and educator rating

	n	Not Adequate	n	Adequate	n	Good/Excellent
All clinical areas combined	31	97%	94	43%	26	88.5
Neurology	5	100%	27	44.4%	9	77.8%
Cardiopulmonary	9	100%	26	46.2%	5	100%
Outpatient Musculoskeletal	6	83.3%	8	33.3%	7	86%
Inpatient Musculoskeletal	11	100%	33	42.4%	5	100%

**Fig. 3:**
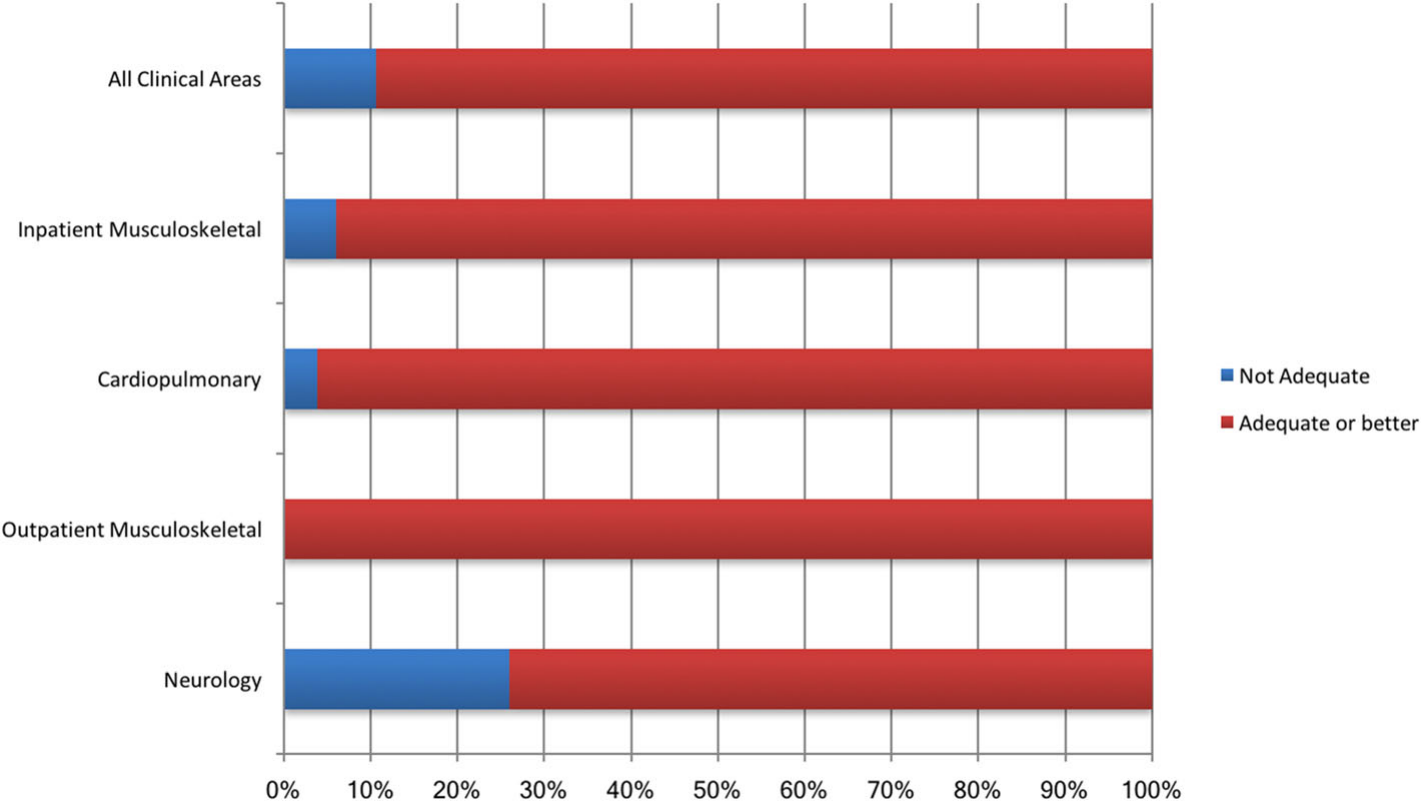
Consensus in Differentiating a ‘Not Adequate’ from an ‘Adequate’ Performance

Consensus between raters in differentiating a performance that meets or exceeds the minimum required standard (i.e. at least an ‘adequate’ performance or better) from a performance below the minimum standard (i.e. ‘not adequate’ performance) was also determined. Outlined in Fig. 3, percent agreement between clinical educators was 89% when differentiating ‘not adequate’ from a performance that meets or exceeds the minimum required standard across all clinical areas. Similarly, across three of the four areas of practice, percentage agreement met the 75% threshold (range 94–100%) when differentiating a ‘not adequate’ performance from an ‘adequate’ or better performance. However, in neurology, this threshold was not achieved and only reached 74% agreement.

### Key behaviours influencing global rating

Thematic analysis of the key behaviours that most influenced participant decision-making is summarised in Table 3. Regardless of clinical area, participants consistently identified similar core attributes that affected their choice in rating the student’s performance. Issues related to risk management (safety) were the most reported behavior that influenced decision-making. Other factors such as technical skill and confidence were also frequently reported as important in decisions related to assessment.

**Table 3:** Key behaviours across all clinical areas that influence assessment decisions using the APP

Professional behaviour	Communication	Assessment	Analysis & planning	Intervention	Risk management
Professionalism throughout assessment Respect Eye contact Privacy	Clear and concise written and verbal communication	Planning, sequence and flow of assessment	Demonstration of clinical reasoning (verbal or written)	Responsiveness to the patient needs	Manual handling skills
Use of informed consent	Promotes rapport	Comprehensive and relevant	Knowledge	Quality of exercise prescription	Awareness of limitations and seeks help
Identification of personal limitations	Recognition of patient factors affecting communication Culturally and linguistically diverse Age Cognition	Inclusion of relevant and appropriate outcome measures	Evidence of a diagnosis, main problem or functional limitation	Reassessment and evaluation of intervention	Body position and ergonomics
	Active listening	Technical and handling skills		Education and explanation for patient and educator	Clear and confident instructions
	Confidence	Awareness of safety issues Red/Yellow flags Investigations Clinical signs		Technical and manual handling skills	Infection control
					Responsive to risk e.g. red/yellow flag and clinical signs and symptoms

## Discussion

Based on the study findings, it appears that physiotherapy educators demonstrate consistency in assessing a student at the ‘not adequate’ and ‘good/excellent’ level regardless of clinical area. However, when assessing the ‘adequate’ performance, educators demonstrated greater variability and lacked consistency based on the parameters of this study (> 75%). Importantly, when adjusted for identifying the ‘not adequate’ performance from the ‘adequate’ or better, educators again demonstrated consistency. This suggests that physiotherapy educators with a minimum of 3 years clinical experience are consistent at ensuring physiotherapy graduates are achieving at least the minimum entry standard during clinical placements based on the APP GRS. However, there is variability in the interpretation of an ‘adequate’ performance.

The ability for a physiotherapy educator to differentiate between a ‘not adequate’ and ‘adequate’ or better performance is a critical decision. The primary objective for assessing student performance in the clinical setting is to determine an acceptable level of professional competence, which aims to minimise risk to the community [10]. Importantly, our results suggest that adequately experienced educators are consistent in their interpretation of the APP GRS when comparing competent and not competent students. In contrast, a similar study by Cross, Hicks et al. [10] reported wide variability in clinical educator interpretation of practice based assessment with a tendency among clinical educators to regress to the mean resulting in a failure to fail unsatisfactory performances. The study also reported that training in assessment improved the consistency [10]. A possible rationale for such a discrepancy is the assumption that most participants had previous training in the use of the APP GRS. Unfortunately, we did not collect data on the level of training undertaken by participants, so it is difficult to draw definitive conclusions on the impact of this on our findings. However, it is common practice among Australian Universities to provide training and support in the assessment and interpretation of the APP to all educators. As a result, this may have influenced the level of consensus observed in this study.

Based on our results, it appears the greatest source of variability occurred when differentiating the ‘adequate’ performance from the ‘good/excellent’. A possible rationale for this finding could be the fact that some components of the student assessment are being performed at either a good/excellent level or in some cases a not-adequate level. The scripts were purposely written with such variability to reflect actual student performance. As a result, an educator’s individual bias may have influenced the final decision of whether to award ‘adequate’ or ‘good/excellent’ when faced with an ultimatum. Analysis of the key behaviours outlined in Table 3 support this assumption, showing that individuals were focused on different aspects of the performance when watching the videos. A similar finding was reported by Trede and Smith [8], concluding that assessment practices among clinical educators were influenced by socio-material structures shaped through experience, indicating that different personal, professional and environmental factors influence decision making when conducting assessment. It is reasonable to conclude that when faced with a difficult decision, such as differentiating between assessment levels on the APP GRS, with limited information, participants within the current study reverted to past experience and context to influence their final decision, which may have resulted in the observed variability. Although in research studies, the influence of such bias is minimized by careful sampling, a lack of consistent training among the sample population may have meant that judgement bias had remained an issue [11, 12].

### Limitations

The primary limitation to the current study was the recognition that the video vignettes created were a one-off student performance lasting on average 20 min. However, in practice the APP instrument was designed to be used for the assessment of student performance during longitudinal clinical placement blocks of 4–6 weeks where student assessment of performance can be done on multiple occasions across time and in a diverse range of patient presentations. Furthermore, it was not practical to include all assessable attributes of student performance that can be observed across time within a single vignette, limiting the assessor’s ability to use a wide range of evidence on which to base their overall rating.

## Conclusions

The study found strong consensus between clinical educators when assessing a ‘not adequate’ and ‘good/excellent’ performance. Furthermore, consensus existed when differentiating a ‘not adequate’ from an ‘adequate’ performance based on the APP GRS. However, variability existed when assessing the ‘adequate’ performance with a lack of consensus in differentiating ‘adequate’ from ‘good/excellent’.

The resources developed for this body of research are freely available online (http://www.appeducation.com.au/videos/2015-video-vignettes.html). In recognition that training can improve consistency in clinical education assessment, it is hoped that the videos provide an evidence based resource for developing educator skills so that “we can progress to calibrated assessors who are able to rate student performance consistent with similarly calibrated colleagues” [13]. Furthermore, strategies to standardise educator training will only serve to greater improve the consistency in the assessment of student performance using the APP and gain consensus among educators.

## Abbreviations

APP: Australian Practice of Physiotherapy
GRS: Global Rating Scale
